# A multi-step approach to overcome challenges in the management of head and neck lymphatic malformations, and response to treatment

**DOI:** 10.1186/s13023-024-03200-2

**Published:** 2024-07-23

**Authors:** Valentina Trevisan, Eugenio De Corso, Germana Viscogliosi, Roberta Onesimo, Alessandro Cina, Marco Panfili, Lucrezia Perri, Cristiana Agazzi, Valentina Giorgio, Donato Rigante, Giovanni Vento, Patrizia Papacci, Filomena Valentina Paradiso, Sara Silvaroli, Lorenzo Nanni, Nicoletta Resta, Marco Castori, Jacopo Galli, Gaetano Paludetti, Giuseppe Zampino, Chiara Leoni

**Affiliations:** 1 Center for Rare Diseases and Birth Defects, Department of Woman and Child Health and Public Health, Fondazione Policlinico Universitario “A. Gemelli” IRCCS, Largo A. Gemelli 8, Rome, 00168 RM Italy; 2 Unit of Otorhinolaryngology, Fondazione Policlinico Universitario “A. Gemelli” IRCCS, Rome, RM Italy; 3 UOC Radiodiagnostica e Neuroradiologia, Dipartimento di Diagnostica per Immagini, Fondazione Policlinico Universitario A. Gemelli IRCCS, Radioterapia, Rome, Italy; 4 Università Cattolica del Sacro Cuore, Rome, RM Italy; 5 Neonatology Unit, Department of Woman and Child Health and Public Health, Fondazione Policlinico Universitario Agostino Gemelli IRCCS, Rome, 00168 Italy; 6 Scuola di Specializzazione in Chirurgia Pediatrica, Università Cattolica Sacro Cuore, Roma, Italy; 7 Department of Biomedical Sciences and Human Oncology (DIMO), Medical Genetics, University of Bari “Aldo Moro”, Bari, Italy; 8 Division of Medical Genetics, Fondazione IRCCS-Casa Sollievo della Sofferenza, Foggia, San Giovanni Rotondo Italy

**Keywords:** Lymphatic malformations, Lymphangioma, Personalized medicine, Sirolimus, mTOR, Alpelisib

## Abstract

**Background:**

Lymphatic malformations are vascular developmental anomalies varying from local superficial masses to diffuse infiltrating lesions, resulting in disfigurement. Patients’ outcomes range from spontaneous regression to severe sequelae notwithstanding appropriate treatment. The current classification guides, in part, clinicians through the decision-making process, prognosis prediction and choice of therapeutic strategies. Even though the understanding of molecular basis of the disease has been recently improved, a standardized management algorithm has not been reached yet.

**Results:**

Here, we report our experience on five children with different lymphatic anomalies of the head and neck region treated by applying a multidisciplinary approach reaching a consensus among specialists on problem-solving and setting priorities.

**Conclusions:**

Although *restitutio ad integrum* was rarely achieved and the burden of care is challenging for patients, caregivers and healthcare providers, this study demonstrates how the referral to expert centres can significantly improve outcomes by alleviating parental stress and ameliorating patients’ quality of life. A flow-chart is proposed to guide the multidisciplinary care of children with LMs and to encourage multidisciplinary collaborative initiatives to implement dedicated patients’ pathways.

## Introduction

Lymphatic malformations (LMs), previously known as cystic lymphangiomas, are benign non-neoplastic vascular lesions stemming from an anomalous embryological development of the lymphatic system. Their estimated incidence ranges from 1 in 6000 to 1 in 16,000 without any sex predilection [1]. LMs might be isolated or associated with other vascular malformations, such as capillary malformations (CMs), venous malformations (VMs), arteriovenous malformations (AVMs) and arteriovenous fistulas (AVFs) [2]. Although LMs might be diagnosed before birth, their usual onset is in early infancy or during the first years of life, with a phenotypic spectrum ranging from mild to severe forms. The most commonly involved areas are the head and neck region, axillae, groins, retroperitoneal tissues, tongue, ​​and mucous membranes of the oral cavity [3]. LMs can be classified into macrocystic, microcystic, and mixed lesions, according to their structure. Macrocystic lesions appear as anechoic lobulated cysts (diameter > 1–2 cm) with multiple septa. In case of infections, that are a common complication of LMs, some proteinaceous material can be recognized inside the cysts. Microcystic LMs are characterized by smaller cysts, with a diameter < 1–2 cm. Since ultrasonographic findings are often nonspecific they can often be misdiagnosed with the only aid of US, therefore MRI might be more accurate. Mixed lesions have characteristics that are ascribable to both macro- and microcystic LMs [2].

According to their location, LMs’ excessive growth can lead to disfigurement, bleeding, organ dysfunction or airway obstruction, especially when located in the head and neck regions [1]. Moreover, sudden growth might be triggered by different stimuli such as infections, worsening the outcomes of affected individuals [4]. The standardized treatment strategies are sclerotherapy for macrocystic LMs, and surgery for microcystic ones [5], but in recent years pharmacologic therapy targeting the PI3K/AKT/mTOR pathway at different levels are emerging [5].

The updated classification of vascular anomalies provided by the International Society for the Study of Vascular Anomalies (ISSVA) [2], includes LMs among vascular malformations [2] and the recent advances in the genetic field have further expanded the molecular landscape of vascular anomalies [6, 7]. Somatic mutational events in LMs frequently involve dysregulation of the PI3K/AKT/mTOR and the RAS/MAPK pathways. LMs are in fact the most common manifestation among the *PIK3CA*-related disorders, and they can be considered as a spectrum of sporadic hamartomatous conditions due to somatic, gain-of-function, deleterious variants in the phosphatidylinositol-4,5-bisphosphate 3-kinase catalytic subunit alpha (*PIK3CA)* gene, which encodes for a key component of the PI3K/AKT/mTOR pathway [8]. On the other hand, both *BRAF* and *NRAS* genes, which are involved in the RAS/MAPK pathway, have been also associated to the molecular blueprint of the condition [9]. According to Zenner et al., the recurrent somatic *BRAF* p.Val600Glu (c.1799T > A) substitution can be identified as the molecular biomarker in approximately in 3% of the cases [10, 11]. Moreover, somatic events involving the *NRAS* gene have been observed in some individuals with kaposiform lymphangiomatosis [12]. Even though some patients remain without an identified molecular signature to support clinicians in their management, the recent improvements in personalized medicine have paved the way to the use of targeted therapies in LM patients who receive a molecular diagnosis [7, 13]. Due to the abovementioned reasons, a standardized management algorithm to guide clinicians through the decision-making process, prognosis prediction, and personalized therapeutic strategies is still evolving from the first proposal from the European Reference Network on Rare Multisystemic Vascular Diseases (VASCERN) - Vascular Anomalies Working Group (VASCA-WG) who recently provided diagnostic, management, and treatment strategies for LMs [14]. Therefore, we herein report our contribution in defining an efficient dedicated care for pediatric patients with LMs while treating five children with LMs, ranging from mild to severe forms.

## Aim, patients and methods

### Objective of the study

The purpose of our study was twofold: (1) to evaluate efficacy of an implemented comprehensive diagnostic, management, and treatment work-up, following the ISSVA and the VASCERN-VASC indications [2, 14] to treat patients with complex LMs (2) to assess LMs patients’ and caregivers stress and psychosocial well-being in the context of complex LMs. We also aimed at gaining in depth knowledge about their experience and their views of the impact of participating.

### Population cohort

**Graph 1:**
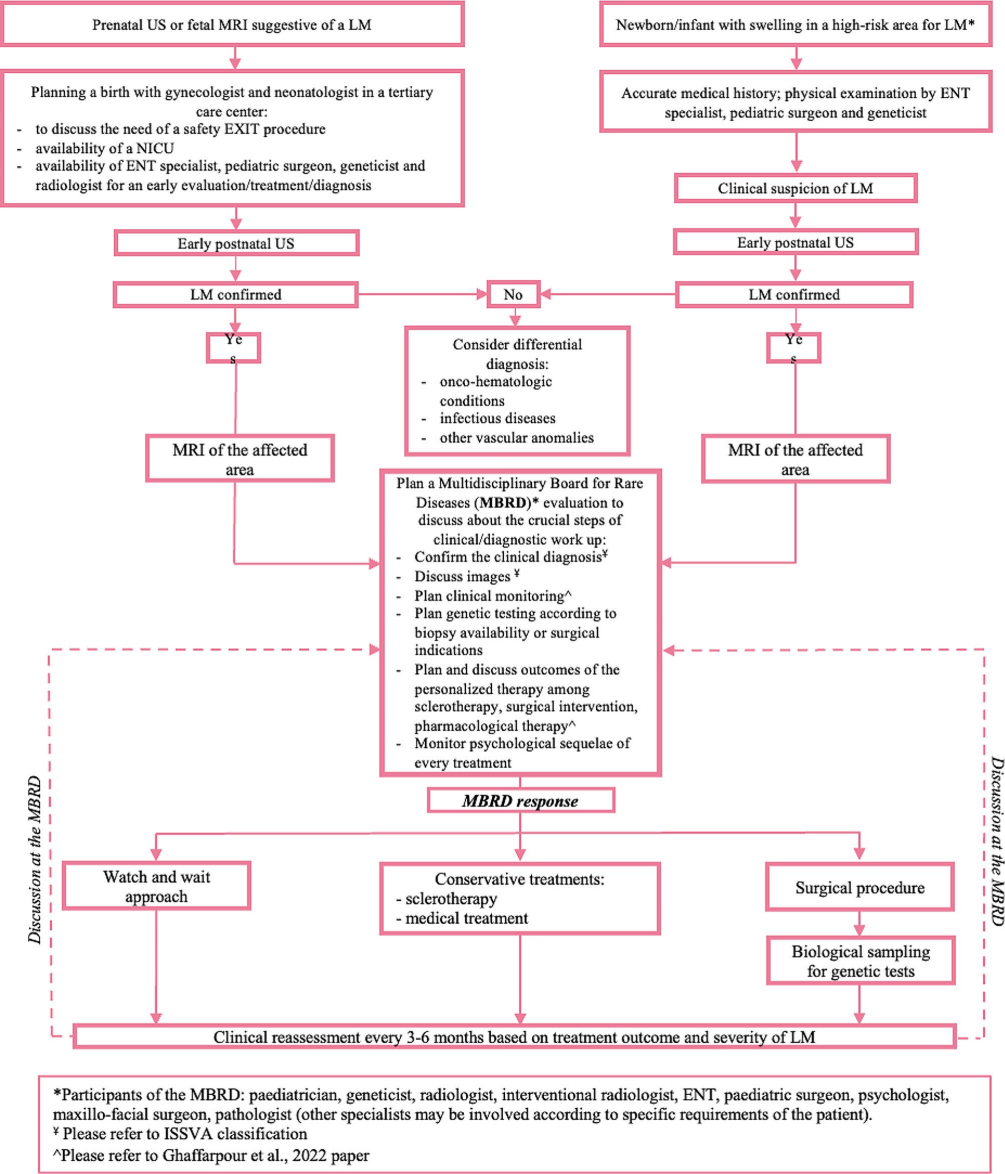
Systematic multistep approach applied to patients with isolated LMs of the head and neck region

We retrospectively analysed a cohort composed by five children with isolated mild-to-severe LMs of the head and neck region all recruited at the Center for Rare Diseases and Birth Defects of our Institution (A. Gemelli University Hospital IRCCS, Catholic University of Sacred Heart, Rome Italy). A systematic multi-step approach was applied to overcome challenges in management (Graph 1). The Nominal Group Technique was used to structure the specialists’ interaction and group discussion in the frame of a Multidisciplinary Board for Rare Diseases (MBRD) with the aim of reaching a consensus, problem-solving, determining priorities, and generating an agreement between specialists upon provided suggestions and recommendations [15]. A comprehensive diagnostic, management, and treatment work-up, following the ISSVA and the VASCERN-VASC indications [2, 14], was discussed and applied for each patient by the MBRD of our Institution, combining it with the large institutional experience on diagnosis, management and therapy for patients affected by different rare diseases/congenital malformations [16–20].

### Assessment procedure

Following the VASCERN-VASCA workflow and the experience of our Institution in the multidisciplinary management of patients with rare diseases, physicians experienced in LMs’ diagnosis examined all individuals and discussed possible treatment strategies in the contest of the MBRD: (i) a pediatrician and a geneticist to assess indication for genetic conditions associated to LMs; (ii) an otolaryngologist to plan surgical procedures and perform a comprehensive ear-nose and throat (ENT) evaluation including a flexible fiberoptic nasolaryngoscopy in case of suspicion of airways localization and potential obstructive problems; (iii) a pediatric surgeon to plan and discuss therapeutic strategies (sclerotherapy vs. surgical excisions); (iv) a radiologist to discuss about the best approach and follow-up frequency, to perform and review the exams; (v) a psychologist to evaluate patients’ and parental stress, to assist families since the diagnosis throughout the therapeutic pathway, to provide support in case of patient’s burnout and parental stress.

In case of biopsy or surgical intervention (with consequent specimen examination) the pathologist provided valuable insights in the histological interpretation of tissue samples, supporting the diagnostic process.

Doppler Ultrasound (US) was the first-line instrumental examination to study the characteristics of LMs (macro-cystic, micro-cystic, or mixed). An abdominal US and cardiac evaluation (EKG and echocardiogram) were performed in all patients at baseline. Magnetic resonance imaging (MRI) study was performed in all individuals at diagnosis and/or before sclerotherapy/surgery treatment to evaluate extension of LMs and involvement of deep-seated anatomical sites, not assessable by US. Complete blood count, coagulation tests (including prothrombin time, activated partial thromboplastin time, fibrinogen and D-dimer), liver/kidney function and thyroid hormones assessment were performed in all individuals before sclerotherapy or surgery.

To assess patients’ and families psychological and social well-being, the “Adaptive Behavior Assessment System (ABAS)”, “Sleep Disturbance Scale for Children (SDSC)” and the “Parenting Stress Index-Short Form (PSI-SF)” questionnaires were administered to all eligible parents and caregivers.

Histological analysis was performed on accessible LMs’ biopsies from patients undergoing surgery. Concerning genetic testing, peripheral venous blood, fresh biopsies, and buccal swab samples were collected for DNA extraction [by using the QIAamp Mini Kit (Qiagen, Hilden, Germany)], quantification [on a Bio Spectrometer Plus (Eppendorf, Hamburg, Germany)] and genetic test. Next-generation sequencing (NGS) analysis was performed on genomic DNA from each sample type with AmpliSeq Illumina Custom DNA Panel (2021 Illumina). The custom panel included the following genes: *PIK3R1, PIK3R2, PIK3CA, PTEN, PDK1, PDK2, KRAS, AKT1, AKT2, AKT3, RICTOR, MAPKAP1, MLST8, MTOR, IRS1, GAB1, GAB2, THEM4, MAPK8I1, PTPN11, RAPTOR, RASA1, TEK, TSC2, GNAQ, TSC1, DEPDC5, CCND2, NPRL3* and *GNA11* [21]. Sequencing runs were carried out according to the manufacturer’s protocol on a MiSeq Instrument (Illumina, San Diego, California, USA). Data analysis was performed using Local Run Manager software V.3 (Illumina). Reads were aligned to the hg19 human reference genome, and alignments were visually verified with the software Alamut V.2.15 (Interactive Biosoftware). In selected cases, the IGV data were scrutinized in mutational hot spots to exclude reads discarded for quality issues. NGS was performed to obtain an average, unique on-target read depths > 1000× to detect variants at very low levels [1% variant allele fraction (VAF) at a depth of 1000×]. Only variants with a VAF > 1% were considered and studied according to different levels of evidence supporting pathogenicity in cases of somatic mosaicism.

The variant call format (VCF) file was first screened for variants previously known as associated with LMs. Subsequently, the annotated variants were filtered with a variant-agnostic approach by using standard procedures for rare Mendelian disorders, which included allele frequency (https://gnomad.broadinstitute.org; i.e. all variants with a minor allele frequency ≥ 0.01 were filtered out), variant type, previous occurrences in public databases (https://www.ncbi.nlm.nih.gov/clinvar/; https://www.ncbi.nlm.nih.gov/snp/; https://cancer.sanger.ac.uk/cosmic) and PubMed, and *in silico* predictions on a consensus of tools including REVEL, combined annotation dependent depletion (CADD) and Polyphen2. Assuming a somatic origin of the candidate variant, genotypes were prioritized if discordant against peripheral blood.

## Results

### Case 1

Case 1 is a girl referred to our attention at 8 days of life (DOL) for the evaluation of a cervical mass of soft consistency. She was born at 40 + 2 weeks of gestational age (GA) by vaginal delivery with unremarkable prenatal history. The father reported personal history of a subcutaneous dorsal cavernous malformation which spontaneously resolved during his first months of life.

**Fig. 1:**
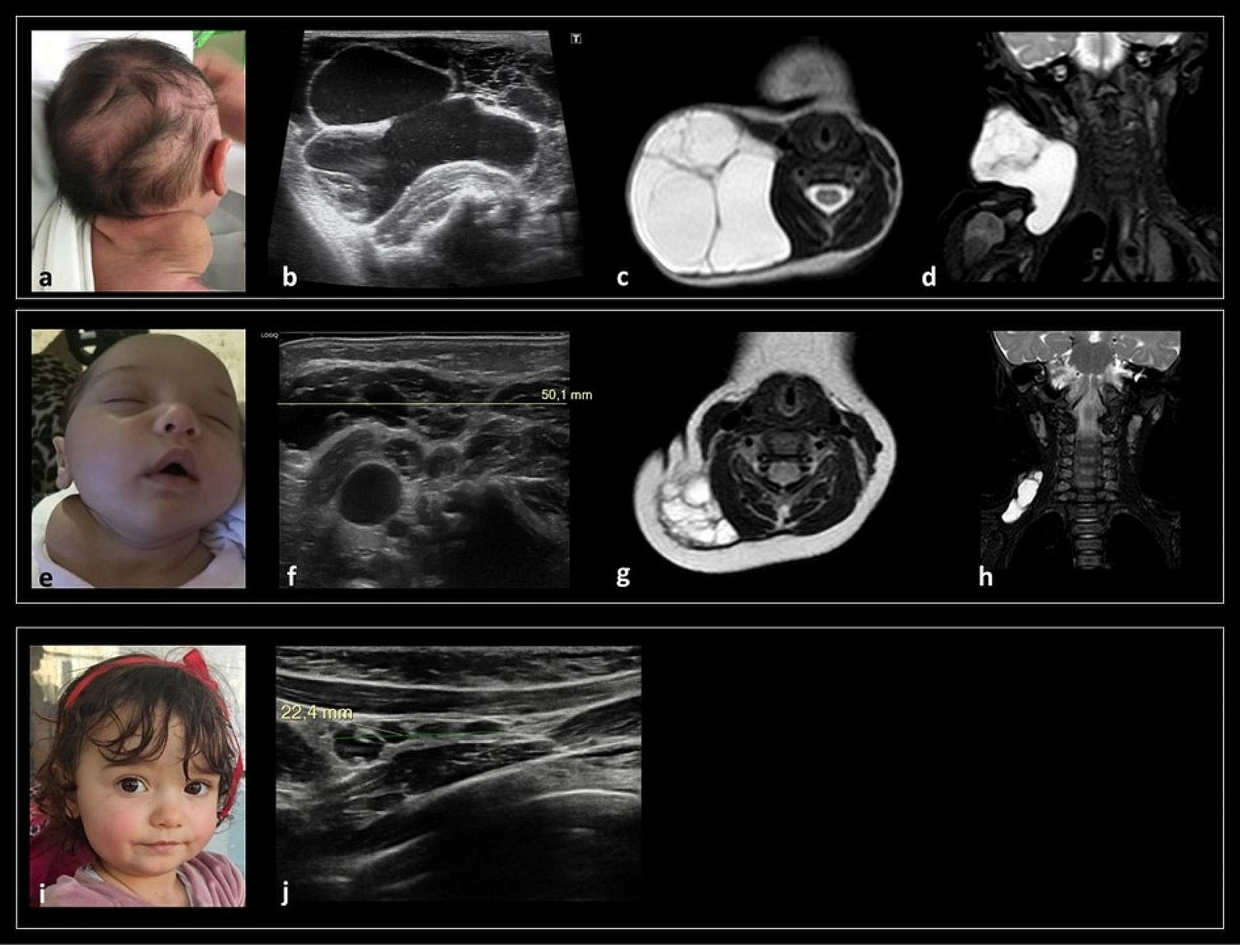
(**a**) Clinical examination at 8 DOL showing a cervical swelling mass, extending from the posterior lateral area of the neck to the ipsilateral supraclavicular region. Pre-treatment US (**b**) and MRI (**c, d** - axial and coronal T2-w respectively) confirmed a lateral cervical multiloculated cystic mass in the posterior lateral triangle of the neck, extending inferiorly in the right infraclavicular space, without involvement of mediastinum and visceral space of the neck. One-month post-sclerotherapy, the patient presented a marked clinical reduction of the cervical mass (**e**). US examination confirmed a dimensional reduction of the lymphatic lesion, with maximal extension of 5 cm towards the deep tissues (**f**). Six months post-sclerotherapy, MRI (**g, h** - axial and coronal T2-w respectively) showed > 50% volumetric reduction with a residual macrocystic mass in the right posterior lateral neck, without infiltration of muscles and deep neck spaces. (**i**) Picture at last clinical evaluation (2 years and 2 months of age) did not document any visible mass, and US examination (**j**) showed resolution, with a residual hypo-/anechoic multiloculated component in the right supraclavicular area (24 × 5 mm)

At birth, due to the evidence of a cervical mass, a karyotype analysis (46, XX), chest and abdominal X-ray and a neck US were performed. The US showed a subcutaneous anechoic multichambered cystic formation with a maximal diameter of 7 cm, located in the right lateral cervical area. The lesion was also appreciated at the first physical examination performed at our Institution as large swelling mass with soft consistency, in the right posterior-lateral area of the neck, extending to the supraclavicular region (Fig. 1a). A further neck US performed in our centre confirmed a multichambered macro-cystic lesion localized laterally to the neurovascular bundle and below the sternocleidomastoid muscle, with diameters of 7.5 × 4.5 cm and a cranio-caudal extension of 5 cm (Fig. 1b). Airways compression and the progression of the LM in the mediastinal region were ruled out by neck MRI (Fig. 1c-d). The MBRD discussion considered the patient eligible for sclerotherapy with OK-432. One month after sclerotherapy (Fig. 1e), neck US revealed a marked dimensional contraction of the lymphatic lesion, with maximal extension of 5 cm towards the deep tissues (Fig. 1f). A gradual volume reduction was evident at the 6 months post-sclerotherapy follow-up, and a volumetric shrinkage of more than 50% compared to the initial dimensions was confirmed by MRI, along with a residual macro-cystic mass in the right posterior lateral neck, without muscle infiltration (CCD 39 mm x APD 38 mm x LLD 16 mm post sclerotherapy vs. CCD 68 mm x APD 63 mm x LLD 46 mm pre-sclerotherapy)(Fig. 1g-h). Regular clinical and US monitoring was performed every 6 months. At her last evaluation (2 years and 2 months of age) (Fig. 1i), clinically the lateral mass was not palpable anymore, and US showed complete resolution of the expansive lateral cervical lesion, with only a residual hypo-/anechoic multiloculated component in the right supraclavicular area (24 × 5 mm) (Fig. 1j). Since no pharmacological therapy was considered necessary, molecular testing was not attempted.

### Case 2

**Fig. 2:**
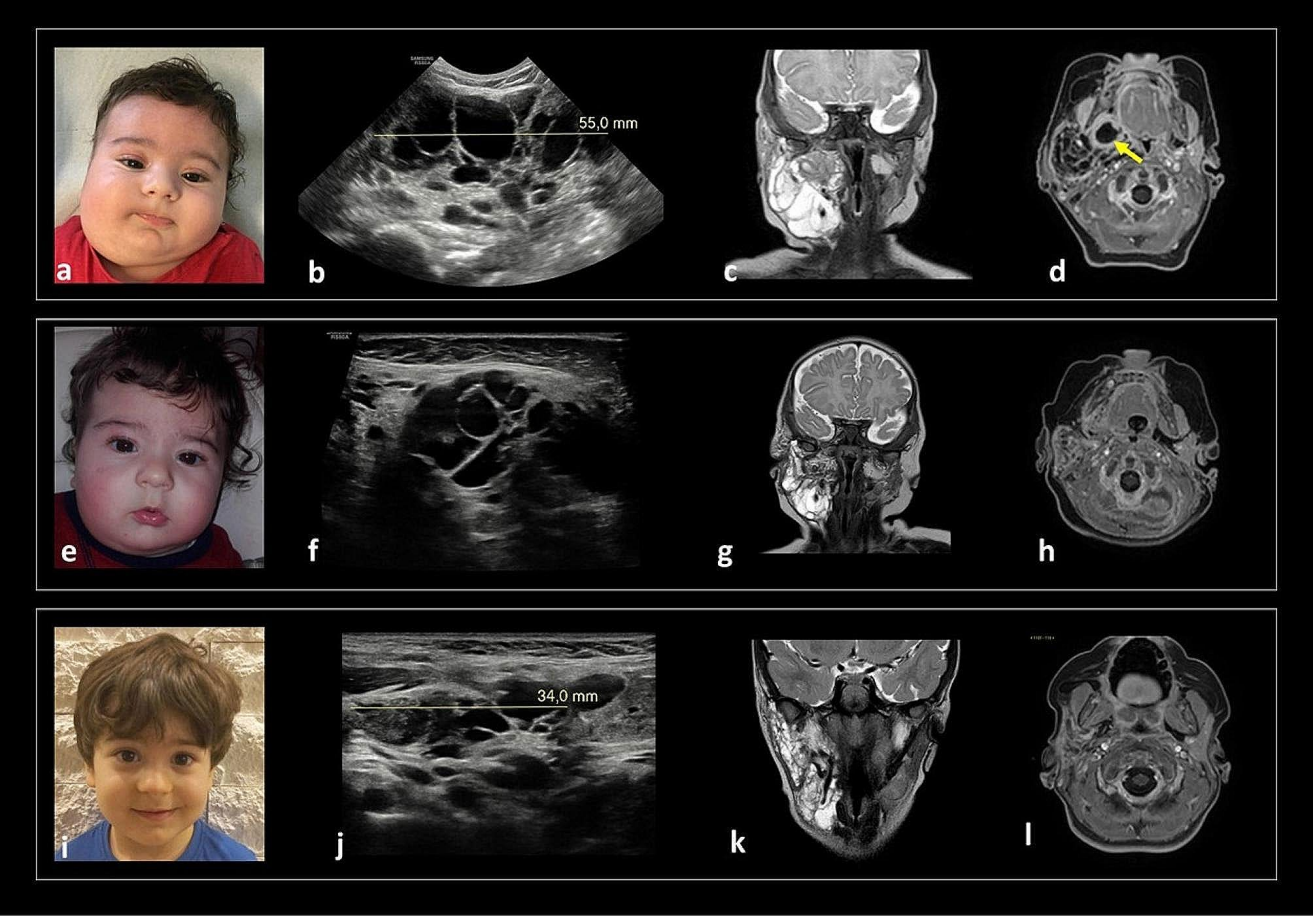
Clinical examination at 40 DOL (**a**), showing a lateral cervical mass on the right side of the face and neck, overlain by hyperaemic and warm skin. US (**b**) and MRI (**c, d** coronal T2w and axial T1-w after contrast medium) confirmed a large, combined LM in the right upper neck with extensive multicompartmental involvement, infiltration of the parotid, masticatory, and parapharyngeal and retropharyngeal spaces; a parapharyngeal abscess (**arrow**, fluid collection with thick peripheral rim of contrast enhancement), compatible with the clinically suspected superinfection, was present. Seven weeks post-sclerotherapy (5 months of age) a clinical improvement of the lesion was detected (**e**). US (**f**) and MRI (**g**: coronal T2w, **h**: axial T1-w after contrast) showed slight volumetric reduction of the LMs. The clinical examination at 2 years and 5 months showed a very mild asymmetry of the right lateral neck (**i**). Concomitant US showed a further reduction of the multi-chambered lesion, from 5 cm to 3.4 cm (**j**). The 3 years follow-up MRI (**k**: coronal T2w, l: axial T1-w after contrast) showed residual microcystic components in the right parotid, parapharyngeal and submandibular spaces

Case 2 is a boy born at 38 weeks of GA by vaginal delivery with uneventful prenatal and perinatal history. He was admitted to the emergency room of our hospital for a febrile episode at 40 DOL, when a soft lateral cervical mass was appreciated at physical examination on the right side of the face and neck, overlain by hyperaemic and warm skin (Fig. 2a). No breathing or feeding concerns were reported by caregivers. Neck US performed at that time (Fig. 2b) showed the presence of a multi-chambered mass (max dimensions 5.5 × 3.2 cm), compatible with a mixed micro/macro-cystic LMs. Complete blood count (CBC) showed increased white blood cells (WBCs) and inflammatory markers, which prompted the introduction of an early intravenous antibiotic treatment. The patient was hospitalized for further evaluations: an abdominal US revealed a round hyperechogenic lesion of 15 mm in the VII hepatic segment, which was confirmed as hepatic angioma by abdominal CT scan. Overall, clinical ENT exam and hearing tests were normal, while overnight pulse-oximetry study detected a decreased level of oxygen saturation (minimal SatO_2_ detected = 89%). MRI study confirmed the presence of a large micro- and macro-cystic LM in the right upper neck with extensive multicompartmental involvement which involved infiltration of the masticator space and parapharyngeal and retropharyngeal spaces, attributable to a parapharyngeal abscess, confirming the suspected infection (Fig. 2c-d).

After evaluation and discussion at the MBRD, the patient was considered eligible for sclerotherapy which was performed on the three largest macro-cysts site at the right submandibular angle at 99 DOL (Fig. 2e). A partial decrease in volume was observed at US performed 7 weeks after the procedure (5 × 2 cm) (Fig. 2f) and MRI (Fig. 2g-h). Clinical and routine US monitoring showed a spontaneous progressive reduction of the residual lesion with max diameter of 40 mm at 17 months. Between 2 and 22 months of age the patient had 5 febrile episodes, without LM localization, all successfully treated with anti-inflammatory therapies (paracetamol, ibuprofen). At 23 months of life, after an infective episode of the upper airways along with fever treated at home with paracetamol, an increase in volume of the caudal component of the mass (35 × 15 mm and craniocaudal diameter 55 mm) was confirmed by US. The oral antibiotic treatment led to improvement of LMs diameter (*restitutio* to pre-infection dimensions). Given the slow evolution of the LMs and the absence of airway obstruction or infiltration of soft tissue or vital organs, the MBRD recommended a “watch and wait” approach with regular US monitoring without further therapy recommendations.

At the last follow-up evaluation performed at 2 years and 5 months (Fig. 2i), the neck US showed a further reduction of the multi-chambered lesion, from 5 cm to 3.4 cm (Fig. 2j). At 3 years, follow-up MRI showed a residual micro-cystic component of the lesion (Fig. 2k-l). As there was no indication for pharmacological intervention, molecular testing was not pursued.

## Case 3

Case 3 is a boy with prenatal diagnosis of a giant cervical and facial mass detected by routine US monitoring at 22 weeks of GA, confirmed by prenatal MRI.

**Fig. 3:**
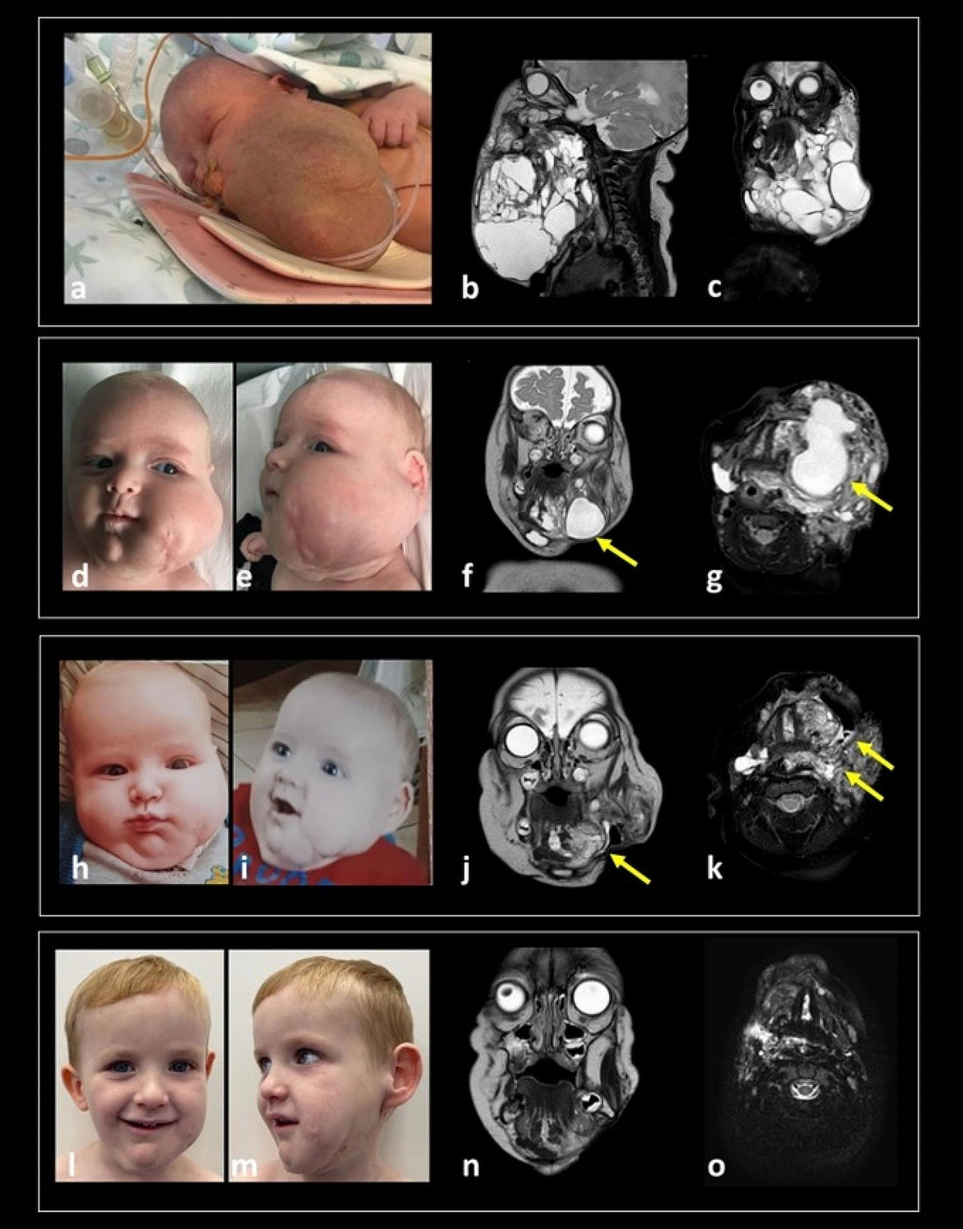
A large mass with elastic consistency involving the neck and the left side of the face was confirmed at birth(**a**). MRI at the time showed a large multicompartmental mixed solid-cystic mass (mixed solid enhancing and cystic non-enhancing components) that involved the left face and the suprahyoid and infrahyoid neck superficial and deep spaces, including parapharyngeal and visceral spaces infiltration (**b, c**). At 3 months of life, a clinical rapid worsening of the swelling mass in the left cheek was detected (**d, e**). A follow-up MRI (**f, g**) confirmed increased volume of some macrocystic components along the left submandibular and parapharyngeal spaces (arrows). After the second surgical procedure, a clinical improvement of the facial asymmetry was evident with reduction of the left swelling cheek mass (**h, i**) and the MRI (**j, k**) showed residual microcystic components in the left sublingual and parapharyngeal spaces (**arrows**). At last evaluation, 4 years 6 months old (**l, m**), head and neck MRI (**n, o**) revealed a reduction of the residual lymphatic tissue located in the left sublingual and submandibular spaces and in the left neck (Left MRI column: sagittal T2-w; Right MRI column: coronal T2-w)

To secure the airways during delivery, the patient was delivered at term in our Institution by C-section and EX-Utero-Intrapartum Treatment (EXIT), with APGAR score 7 = 1’ and 8 = 5’and birth weight of 4.1 kg (87th percentile). For general monitoring, he was admitted to the Neonatal Intensive Care Unit (NICU), where a large mass with elastic consistency involving the neck and the left side of the face was confirmed and evaluated (Fig. 3a). Cardiac US showed patent foramen ovale (PFO), while brain and abdominal US, ophthalmologic and hearing evaluations, clinical ENT, and endoscopy were all normal. Head and neck US and MRI performed soon after birth showed a large multi-compartmental mixed venous and micro- and macro-cystic LM that involved the left face and the suprahyoid and infrahyoid areas of the neck, with parapharyngeal space infiltration and dislocation of the pharyngo-laryngeal air column without causing obstruction (Fig. 3b-c). After the MBRD discussion, the patient was considered eligible for surgical debulking which was performed at 6 DOL. A surgical excision of the complex cervical facial lesion was performed with an open latero-cervical approach, without the need of a tracheotomy. The skin excess was removed through plastic resurfacing and the post-operative period was uneventful. Peripheral venous blood, fresh biopsies and buccal swab samples were collected for genetic testing (see above). Histological evaluation confirmed a giant venous-lymphatic vascular malformation, whereas genetic testing turned out not informative. Post-surgical MRI showed significant mass reduction and improvement of the airways’ dislocation.

A worsening of the left cheek’s LM was detected at three months of life with swelling (Fig. 3d-e), warmth and redness of the overlying skin, along with mild fever and increased WBCs. The MRI confirmed a worsening of the LM characterized by a volumetric increment (52 mm APD x 26 mm LLD x 50 mm CCD) of the macro-cystic component along the left submandibular and parapharyngeal spaces (Fig. 3f-g). While the patient was hospitalized receiving an empiric antibiotic therapy, the MBRD provided the indication to a second surgical procedure. At 3 months and 23 DOL, a second partial surgical excision was performed. Seven days after surgery, a reduction of the residual LMs was appreciated by clinical examination (Fig. 3h-i), the MRI showed some residual micro-cystic components (Fig. 3j-k). After discussion, the MBRD members suggested to start off-label therapy with Sirolimus at 0.8 mg/kg every 12 h. A comprehensive disease reassessment with clinical evaluation (anthropometric measurements, pediatric, ENT, neurological evaluations), laboratory and US monitoring was performed every 3 months in the first year of life. During follow-up visits, a moderate right posterior plagiocephaly associated to visual function immaturity was detected and required physical therapy for 8 months. The growth parameters and the eating abilities were always considered appropriate with no signs of airways’ obstruction. Thereafter, considering the clinical and radiological stabilization of the LM, the follow-up was performed every 6 months. The clinical examination performed at 1 year of age detected an overall stable facial asymmetry, and the MRI study showed a further reduction in the LM.

During the overall treatment period with sirolimus (from 6 months of age to 4 years and half), 10 episodes of mild infections that spontaneously resolved were recorded, and 6 further episodes requiring antibiotic therapy (mostly otitis media) without sequelae were treated.

At last evaluation, (4 years 6 months old), a persistent asymmetry of the face was present; the soft consistency of the skin raised the hypothesis of left soft tissue hemihypertrophy (Fig. 3l-m) with no worsening of the LMs mass. Head and neck MRI revealed a reduction of the residual lymphatic tissue located in the left sublingual and submandibular spaces and in the left neck with almost complete resolution of the pharyngo-laryngeal submucosal and sublingual components (Fig. 3n-o).

## Case 4

**Fig. 4:**
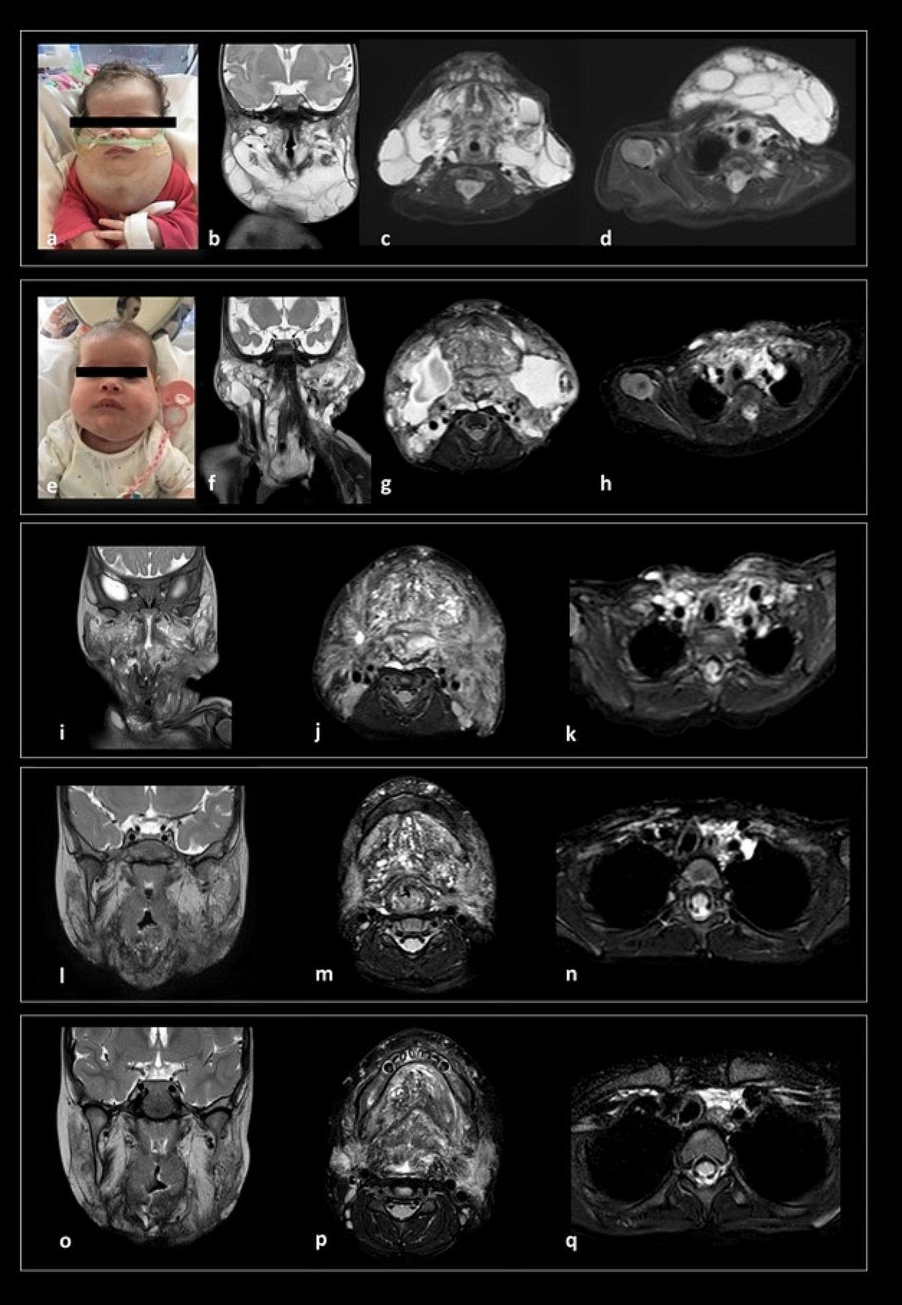
(**a**) Picture at birth showing a large soft swelling involving the neck, with tongue hypertrophy. MRI after birth (**b, c,d**) showed a voluminous macro- and microcystic LM involving the tongue, and with bilateral extensive involvement of the suprahyoid spaces (sublingual, submandibular and parotid spaces) and caudal spread up to the upper mediastinum, along with the parapharyngeal, retropharyngeal, vascular and visceral neck spaces. Persistent swelling of the neck was evident soon after surgery and sclerotherapy (**e**). MRI study (**f, g, h**) performed 10 days after these procedures documented a mild enlargement of the LM in the deep spaces of the neck and in the upper mediastinum, with microcystic involvement of the tongue and oropharyngeal walls causing pharyngeal air column obstruction needing tracheostomy placement. An increased volume of superficial macrocystic components was also detected. The serial follow-up MRI scans during sirolimus therapy (7 months 22 day – **i, j, k**, 2 year 8 months – **l, m, n**, 6 years **– o, p, q)** show progressive volumetric reduction of the LMs, especially the upper mediastinal components (left column, coronal view, T2-w; middle and right column, axial view, T2-w fat sat) respectively passing through the oropharynx and the upper mediastinum. The last US examination (**j**) shows absence of residual macrocystic components in the superficial neck spaces

Case 4 is a girl born at our Institution by cesarean section with EXIT procedure planned at 37 + 2 weeks of GA due to a prenatal diagnosis (US and MRI study) of a large cervical mass involving the neck, the tongue, and extending to the apex of the left lung, suspected to be a LM. At birth, the patient was intubated and admitted to the NICU for clinical monitoring. A large soft swelling involving the neck was evident in association to tongue hypertrophy (Fig. 4a), without any other related malformation. Head and neck US and MRI studies performed after birth showed a voluminous mixed cervical LMs with bilateral extensive involvement of the suprahyoid and infrahyoid neck spaces (sublingual, submandibular and parotid spaces), involvement of the base of the tongue, and caudal spread up to the upper mediastinum along the parapharyngeal, retropharyngeal, vascular, and visceral neck spaces (Fig. 4b-d). During hospitalization, brain MRI, abdominal US, eye examination and cardiac US resulted normal. After multidisciplinary discussion at the MBRD, the child underwent sclerotherapy with OK-432 and surgical biopsy at 15 DOL (Fig. 4e). Biological samples were sent for genetic testing which resulted negative. Histological exam confirmed a venous-lymphatic vascular malformation. An US performed due to the persistency of the large swelling of soft consistency of the neck showed an increased diameter of the mass. A second attempt of sclerosis of the larger cysts with OK-432 was performed at 1 month of age with unsuccessful results. In the following weeks, concomitantly to a progressive incrementing dimension of the superficial and deep cervical lesions, the patient experienced the first respiratory difficulties, and she lost the ability to feed herself. For these reasons, the MBRD opted for a large cervical debulking of the cervical lesions plus an endoscopic laser resection of the prominent base of the tongue, which was performed at 2 months of age, along with placement of a perioperative tracheostomy. To minimize the risk of aspirations, and to improve the growth pattern, a g-tube was placed soon after. The MRI study performed 10 days post-surgery showed a mild progression of the LM in the deep spaces of the neck, the tongue, the midface structures, the superior mediastinum, as well as an enlargement of the superficial cysts of the neck (Fig. 4f-h). The MBRD finally considered the patient eligible to start off-label therapy with sirolimus. Clinical, laboratory and radiological monitoring every 3 months was performed till the first year of life and every 6 months thereafter. During follow-up visits, growth parameters and neuromotor development were normal except for speech delay which required speech therapy support. The clinical examination showed a stable soft tissue hypertrophy mostly located in the cervical region (persistent residual LMs in the parotid spaces, subcutaneous tissue of the cheeks and submandibular spaces) and in the airways, including the base of tongue. Fibro-laryngoscopy and MRI follow-up at 3 years of age confirmed a residual hypertrophy of the base of tongue occluding the laryngeal vestibule, persistent residual LMs in the submucosal oropharynx, parotid spaces, subcutaneous tissue of the cheeks and submandibular spaces, and caudal spread up to the upper mediastinum along the parapharyngeal, retropharyngeal, vascular and visceral neck spaces, with a dynamical circumferential reduction of the oropharyngeal tract, leading to a severe reduction of air space. Furthermore, the swallowing function was not recovered and for that reason the need of tracheostomy tube and G-tube was confirmed. During overall sirolimus therapy, no major adverse events related to the treatment were recorded, except for 6 spontaneously resolved episodes of mild infections (mostly gastroenteritis), and 13 bacterial infections (4 otitis media, 3 pharyngitis, 4 lower respiratory tract infections and 2 urinary tract infections) requiring antibiotic therapy at home (Fig. 4i-q). Temporary drug discontinuation was necessary at 2 years and 6 months of age due to evidence of herpetic gingivostomatitis, associated to feeding difficulties, requiring hospitalization. After complete recovery, sirolimus therapy was resumed at the same dosage. Given the improvement of oral motor abilities, after experts’ consensus at the MBRD, and after complete rehabilitation of dysphagia, the g-tube was removed at 3 years 6 months of age. At her last follow-up (4 years and 6 months of age), a clinical ENT examination with sleep endoscopy showed persistence of severe airway obstruction during sleep. The last MRI showed a progressive volumetric reduction of the LM.

### Case 5

**Fig. 5:**
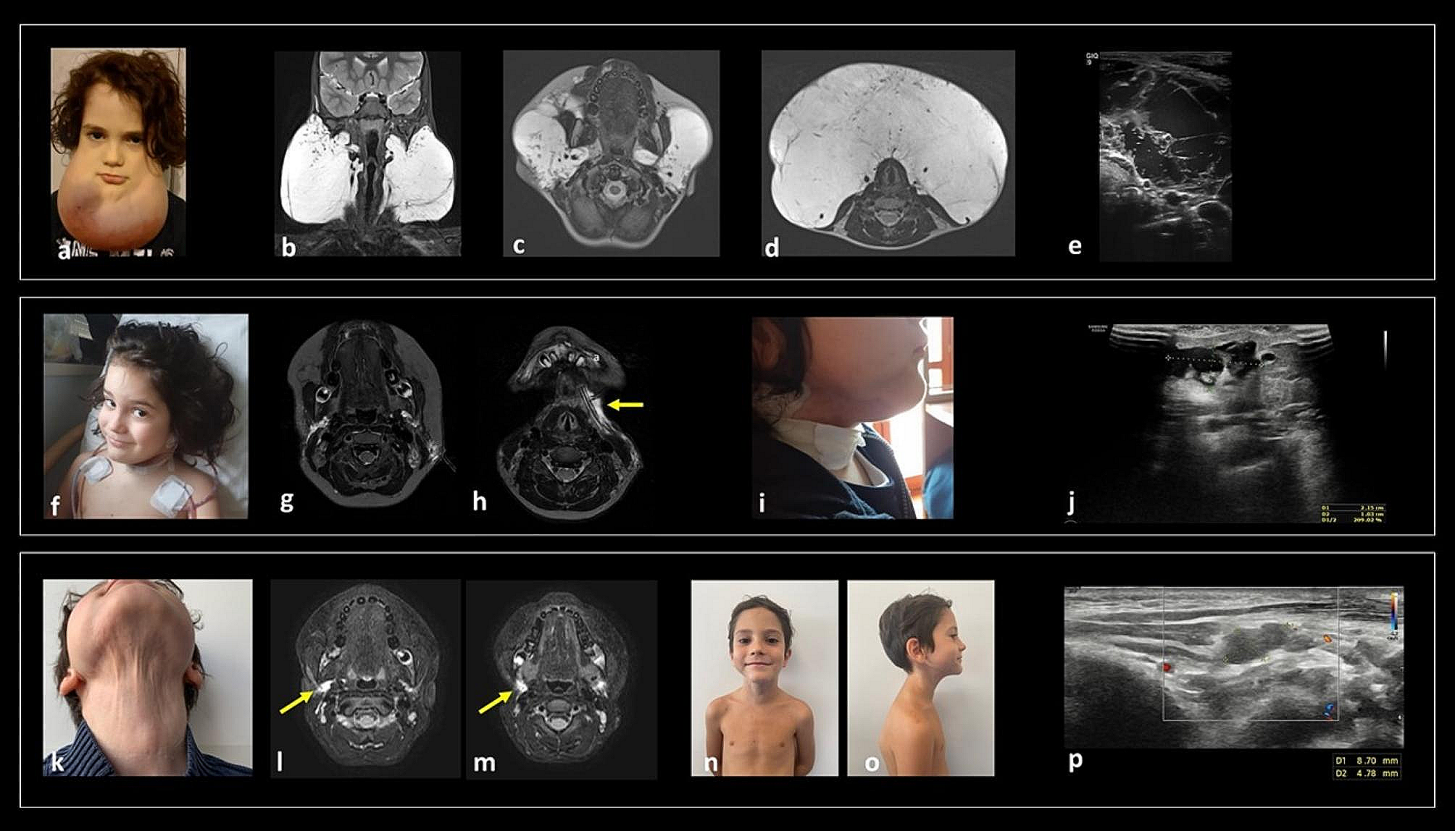
A giant swelling of a soft-elastic consistency originating from the subcutaneous tissues of the mandibular angles bilaterally with downward extension anteriorly to the neck was evident at 6 years and 4 months. The overlying skin showed clinical signs of infection (**a**). MRI (**b-d**) and US (**e**) revealed a large bulky macro-cystic mass in the suprahyoid and infrahyoid neck involving bilaterally the submandibular, parapharyngeal, parotid and vascular spaces, without mediastinal extension and determining minimal deformation of pharyngeal air column, which maintained regular patency. After surgical debulking, the clinical examination revealed an apparent complete response to treatment (**f**). MRI showed grossly total excision of the LM with minimal fluid collections in the submandibular spaces (**g, h**). The arrow indicates the left drainage tube in the submandibular space. Swelling in the retromandibular right angle and submental region was progressively documented after removal of drains (2 weeks post-surgery) (**i**, lateral view). The US performed at that time revealed slight enlargement of some cystic components in the submandibular and submental spaces (**j**). Clinical evaluation at last follow-up (during Alpelisib treatment at 125 mg/day) documented no evidence of swelling mass; the skin in the submental area and in the superior part of the neck appeared thin but not atrophic (**k**). Frontal (**n**) and lateral (**o**) view of the patient confirmed the absence of persistent swelling. The clinically detected facial asymmetry involved the subcutaneous tissues, probably because of the surgical procedure. MR (**l, m**) and US (**p**) showed small residual cystic component in the posterior portion of the right submandibular space (arrow in **l, m**)

The patient is a boy born at term by natural delivery with an unremarkable prenatal and perinatal history. At 5 years of age, apparently after a jellyfish sting, a swelling mass appeared to the right side of lower lip. Soon after, a rapidly growing soft swelling involving subcutaneous tissues of the submental region was noticed. The US study documented a total submental development of a LMs arising from the bilateral subauricular regions with superficial microcystic components in the left submandibular space. Two sclerotherapy treatments with 3% Polidocanol were performed without any clinical benefit. At 5 years 8 months and at 6 years of age respectively, two moderate/severe LMs infection required hospitalization for intravenous antibiotic and corticosteroids therapy administration. At 6 years and 4 months of age the patient was admitted to our hospital for a second opinion. At that time a giant swelling of a soft-elastic consistency originating from the subcutaneous tissues of the mandibular angles bilaterally and extending downward anteriorly to the neck was clinically evident. The overlying skin was red and warm; small wounds draining serous-blood material were also present (Fig. 5a). MRI (Fig. 5b-d) and US (Fig. 5e) of the lesion revealed a large bulky macro-cystic mass in the suprahyoid and infrahyoid neck involving bilaterally the submandibular, parapharyngeal, parotid and vascular spaces, without mediastinal extension. The MBRD shared consensus to surgical treatment. An almost complete excision of the latero-cervical and submental lesion was performed with an open latero-cervical approach after right exofacial parotidectomy with preservation of the facial nerve. The excess skin was removed and a cervicofacial plastic reshaping was performed. To avoid potential nerves’ injury, a residual LM tissue in the upper portion of deep parapharyngeal space deeply to parotid gland was not removed. Tissue samples were collected for molecular analysis and bilateral cervical drainages were placed (Fig. 5f-h). Histological and immunohistochemical analysis confirmed a LM. Considering the progressive reduction of blood-serous fluid, drainages were removed 2 weeks after surgery and the patient was discharged 21 days after the procedure. One week later (28 days from surgery), a clinical reassessment of the child documented a soft distension at the level of the retromandibular right angle and submental region (Fig. 5i). US study confirmed a slight enlargement of some LM’s cystic components in the submandibular and submental spaces (Fig. 5j). MBRD considered the patient eligible to off-label pharmacological treatment with Sirolimus which was started at a therapeutic serum range. Due to a swift worsening of the subcutaneous swelling, a right lateral cervical drainage was placed, and a daily drainage (120–130 ml) of serous-blood material was collected for two following months. Neither clinical nor laboratory markers of infections were detected. The biochemical examination of the drained serum showed high level of amylase (319 UI/l; normal internal laboratory value < 107 UI/l). MBRD approved one botulin injection at the residual right parotid gland, which reduced the daily serum drainage to 80 ml. However, due to persistency of fluid discharge, the drainage was replaced with a long-term one to prevent the accumulation of lymph in the neck. At three months post-surgery, the genetic analysis showed an heterozygous c.1258T > C (p.Cys420Arg) variant in the *PIK3CA* gene in the LM’s tissue sample with a frequency of 9%, not identified in the DNA extracted from the buccal swab, which resulted negative. Hence, considering the persistence swelling in the submental area and the daily drainage, the MBRD recommended wash out from sirolimus therapy and initiation of a PI3K inhibitor. After approval from local EC and signed informed consent by the family, therapy with alpelisib, a selective and orally active PI3Kα inhibitor, was initiated at a dosage of 50 mg/day. A progressive drainage reduction was documented, reaching 60-40 ml per day at 6 months of observation. However, given the persistence of mild subcutaneous swelling clinically evident and confirmed by US monitoring, a unilateral cervical drainage was left in place. MBRD suggested a second injection of botulinum which was performed through US guiding. Moreover, since alpelisib was well tolerated and no major side effects were recorded, the patient was considered eligible for dose escalation to 125 mg/day at 6 months from the alpelisib initiation. This treatment regimen led to a complete remission of drained fluid and allowed removal of the drains 9 months after surgery. Six months after dose escalation, the child showed no evidence of soft tissue masses (Fig. 5k); the skin in the submental area and in the superior part of the neck appeared thin, the frontal (Fig. 5n) and lateral (Fig. 5o) view of the patient documented a facial asymmetry of the subcutaneous tissues, with no evidence of bone remodelling at MRI. A minute residual cystic component in the posterior portion of the right submandibular space was appreciated by MRI and US study (Fig. 5l, m, p). Since the excellent patient outcome with alpelisib therapy, no CT scan of the mandibular bone was recommended. During all pharmacological treatment, the serum glucose level ranged between 78 and 112 mg/dl (expected side effect related to the drug), being only documented adverse event since alpelisib escalation. Given the clinical and radiological disease stability, a dose reduction till 50 mg/day of alpelisib was performed for the following 6 months.

### Psychological assessment

The quality of life and sleep parameters were studied by administering the “Adaptive Behaviour Assessment System (ABAS)”, “Sleep Disturbance Scale for Children (SDSC)” at last evaluation in the entire sample. Results showed a homogeneous and consistent scoring within the normal range, notwithstanding the different patients’ clinical presentations. On the other hand, the parental stress assessment (“Parenting Stress Index-Short Form -PSI-SF-”) showed inhomogeneous results among different families, ranging from values indicating an elevated parental stress (70° centile) to very low values (15° centile). Beside personal coping mechanisms, parental stress levels were skewed to patients’ management complications and intrinsic clinical complexity.

Even though the small cohort and the heterogeneous age group might be considered a limitation for this study, data strongly suggest the need of a prompt introduction of a psychological support in the families of children affected by LMs, especially in the most severe cases, to face patient’s burnout and parental stress. Therefore, the psychologist should be actively involved in the MBRD decision-making process, assisting the family from the communication of the diagnosis throughout the whole therapeutic pathway and subsequent follow-up.

## Discussion

LMs are rare congenital anomalies of the lymphatic system [22] with a heterogeneous etiology and whose molecular mechanisms still need to be unravelled [22]. Given the highly variable clinical presentation, consensus guidelines for their management and treatment are lacking. Unfortunately, conventional treatments are invasive, expensive for National Health Systems, and their outcomes, both aesthetic and functional, are not always optimal, reducing the quality of life of affected individuals [5]. The lack of a standardized algorithm of care for LMs often leads to divergent opinions from appointed clinicians, delaying a timely diagnosis and a tailored treatment. Considering that the usual involvement of facial structures often limits the psychosocial interactions, the lack of a coordinated management worsens the caregiver/parental stress and amplifies the negative impact of the disease on the quality of life of the patients and families. Our study showed that an early psychological support for patients and family enhanced parents’ collaboration and alleviated patients stress levels.

Therefore, a multi-step care approach shared by multidisciplinary experts in LMs is needed to define a more efficient, comprehensive, and personalized strategy for each patient achieving the best patient’s outcome and reducing caregivers’ stress [23, 24]. With the main aim of preserving functionality and anatomical integrity of the affected area as much as possible, the VASCERN/VASCA working group has recently delineated for the first time an efficient diagnostic and management pathway for LMs [14, 25].

Considering available treatment strategies, macrocystic LMs are commonly treated with intralesional sclerotherapy as first line approach, and surgical excision in case of 3 to 4 failed attempts of sclerotherapy [26]. OK-432, bleomycin, and doxycycline are the preferred sclerotizing agents [27, 28]. Recent studies also demonstrated beneficial effects of Polidocanol microfoam [29]. Major complications due to sclerotherapy are the deposits of scar tissue and swelling, creating a mass effect [1, 4, 30]. The management of microcystic/mixed LMs, especially if localized in the head and neck areas, is more complex and may be highly challenging [31]. In such cases, due to the high risk of airways obstruction, a surgical approach is usually preferred, even considering that, due to the intrinsic infiltrative nature of LMs, post-surgical complications such as disfigurement, vascular and nerve damage, bleeding and respiratory failure might occur [30, 32].

Based on our excellent outcomes, the present study demonstrates how a multidisciplinary assessment, through a multi-tier approach based on MBRD, can be applied to tailor treatment strategies for children with mild to severe LMs of head and neck district. As first step to guide management, a molecular screening should be performed at early diagnostic stages or as soon as the LM’s tissue is accessible. As demonstrated by our experience, early detection of a targetable molecular biomarker (e.g. a somatic *PIK3CA* deleterious variant) might lead to better patients’ outcomes compared to a standardized therapy. Recently, also in the context of LMs, the use of repurposed drugs targeting the PI3K/AKT/mTOR pathway at different levels has emerged [5]. The first allocated drug for this purposed was sirolimus (Rapamycin, mTOR inhibitor), which has been used for the treatment of macrocystic, microcystic and mixed lesions, also in the absence of underlying mutations with beneficial results in reducing the volume of LMs [22, 25, 33–35]. However, drug discontinuation has been reported due to severe side effects [4, 36, 37]. Therefore, further multicentric studies/clinical trials are needed to (i) define the LMs patients’ subgroup who benefit from sirolimus therapy also based on LMs’ characteristics, (ii) determine proper therapeutic ranges, and (iii) delineate potential treatment risks and side effects to improve compliance and the overall outcomes [38].

Since somatic *PIK3CA* deleterious variants have been demonstrated to be the most common cause of both isolated and syndromic LMs [8], also other downstream inhibitors of the pathway have been candidate for repurposing [39]. Alpelisib, an inhibitor of p100α catalytic subunit of PI3K, has been successfully used in other PIK3CA-related conditions with lymphatic involvement without evidence of major toxicities [40–44]. Inhibitors of AKT such as miransertib are also being investigated [1, 30]. Lately, the identification of the well-known oncogenic *BRAF* c.1799T > A p.Val600Glu variant on endothelial cells of LMs has paved the way to new therapeutic opportunities like tramentinib targeting the RAS/MAPK pathway [7, 11].

In case of symptomatic, progressive, cystic LMs that do not respond to conventional therapies, the Italian guidelines for vascular anomalies recommend systemic therapy with sirolimus (0,8 mg/m^2^ BID with a therapeutic serum range between 5 and 15 ng/ml) with routine clinical and hematological monitoring to oversee for common adverse events [26]. The VASCERN/VASCA also suggests the implementation with new treatment options (such as PIK3CA-inhibitors) in cases of sirolimus treatment’s failure in patients with a recognized pathogenic variant [45]. The group also stressed the importance of sharing additional research results to define treatment duration, long-term outcome, and potential side effects [14]. Recently, it has also been observed that paracrine vascular endothelial growth factor C (VEGF-C) signalling between mutant and normal endothelial cells contributes to the development of LMs [46]. Studies on mice showed that the combination of mTOR and VEGF-C signalling inhibitors might be more effective in reducing LMs size than sirolimus alone, setting a milestone for future drugs combination [47].

As literature shows, molecular profiling is trivial to tailor treatment [7]. However, due to the deep location of LMs and the high risk of bleeding during a biopsy, genetic testing is not always doable and the molecular fingerprint of LMs remains obscure in many cases [7]. In fact, molecular profiling to date is performed in selected cases with severe phenotypes (such as our cases 3, 4 and 5) who require surgical procedures which enables tissue collection. Moreover, even in the case of surgical indications, molecular profiling by current NGS technologies may be hampered by technical and biological limitations, such as LM’s characteristics, DNA quality, and detection of low level of mosaicism [7].

In our experience, in case of non-responding LM patients, (case 5) the early identification of a molecular target was crucial to choose an alternative therapy. It is therefore fundamental to improve diagnostic techniques and bio-informatic pipeline to identify actionable variants in DNA extracted in biopsies specimen or in liquid biopsies, even at low level mosaicism. Recently, the analysis of cell-free DNA (cfDNA) in patients with kaposiform lymphangiomatosis has enabled for the first time the identification of genetic variants, endorsing the introduction of non-invasive diagnostic techniques [7, 48]. Future studies to address genotype-phenotype correlations are expected to support and develop management updated algorithms and personalized follow-up strategies for children with LMs. Furthermore, future clinical trials with repurposed drugs might provide knowledge based on the long-term efficacy, to design and implement management and treatment protocols.

## Conclusion

In summary, diagnosis, and treatment of LMs of head and neck district are still challenging for clinicians and such lesions remain life-threatening for patients. A psychological support for patients and caregivers is highly recommended to improve parents’ compliance and address the expected challenges in patients’ changes of body image perception. As demonstrated in the present paper, referral of LMs patients to tertiary centres where a multidisciplinary team can develop personalized treatments and prompt molecular identification should be prioritized whenever possible.

## Data Availability

Data sharing is not applicable since no datasets were generated or analysed during the current study. Raw genetic data and further photographic materials are not publicly available to preserve individuals’ privacy under the European General Data Protection Regulation.

## Abbreviations

ABAS: Adaptive Behavior Assessment System
AVFs: Arteriovenous fistulas
AVMs: Arteriovenous malformations
CADD: Combined annotation dependent depletion
CBC: Complete blood count
cfDNA: Cell-free DNA ()
CMs: Capillary malformations
DOL: Days of life
ENT: Ear-nose and throat
EXIT: EX-Utero-Intrapartum Treatment
GA: Gestational age
ISSVA: International Society for the Study of Vascular Anomalies
LMs: Lymphatic malformations
MBRD: Multidisciplinary Board for Rare Diseases
MRI: Magnetic resonance imaging
NGS: Next-generation sequencing
NICU: Neonatal Intensive Care Unit
PFO: Patent foramen ovale
PIK3CA: Phosphatidylinositol-4,5-bisphosphate 3-Kinase catalytic subunit alpha
PSI-SF: Parenting Stress Index-Short Form
SDSC: Sleep Disturbance Scale for Children
US: Doppler Ultrasound
VAF: Variant allele fraction
VASCA-WG: Vascular Anomalies Working Group
VASCERN: European Reference Network on Rare Multisystemic Vascular Diseases
VCF: Variant call format
VEGF-C: Vascular endothelial growth factor C
VMs: Venous malformations
WBCs: Detected increased white blood cells
